# Clustering analysis revealed the autophagy classification and potential autophagy regulators' sensitivity of pancreatic cancer based on multi‐omics data

**DOI:** 10.1002/cam4.4932

**Published:** 2022-06-09

**Authors:** Yonghao Chen, Jialin Meng, Xiaofan Lu, Xiao Li, Chunhui Wang

**Affiliations:** ^1^ Department of Gastroenterology West China Hospital of Sichuan University Chengdu Sichuan P.R. China; ^2^ Department of Urology, The First Affiliated Hospital of Anhui Medical University Hefei P.R. China; ^3^ Institute of Urology Anhui Medical University Hefei P.R. China; ^4^ Anhui Province Key Laboratory of Genitourinary Diseases, Anhui Medical University Hefei P.R. China; ^5^ State Key Laboratory of Natural Medicines, Research Center of Biostatistics and Computational Pharmacy China Pharmaceutical University Nanjing P.R. China

**Keywords:** autophagy, bioinformation, drug sensitivity, multi‐omics, pancreatic carcinoma

## Abstract

**Background:**

Pancreatic ductal adenocarcinoma (PDAC) is a lethal malignancy and is unresponsive to conventional therapeutic modalities due to its high heterogeneity, expounding the necessity, and priority of searching for effective biomarkers and drugs. Autophagy, as an evolutionarily conserved biological process, is upregulated in PDAC and its regulation is linked to a poor prognosis. Increased autophagy sequestered MHC‐I on PDAC cells and weaken the antigen presentation and antitumor immune response, indicating the potential therapeutic strategies of autophagy inhibitors.

**Methods:**

By performing 10 state‐of‐the‐art multi‐omics clustering algorithms, we constructed a robust PDAC classification model to reveal the autophagy‐related genes among different subgroups.

**Outcomes:**

After building a more comprehensive regulating network for potential autophagy regulators exploration, we concluded the top 20 autophagy‐related hub genes (GAPDH, MAPK3, RHEB, SQSTM1, EIF2S1, RAB5A, CTSD, MAP1LC3B, RAB7A, RAB11A, FADD, CFKN2A, HSP90AB1, VEGFA, RELA, DDIT3, HSPA5, BCL2L1, BAG3, and ERBB2), six miRNAs, five transcription factors, and five immune infiltrated cells as biomarkers. The drug sensitivity database was screened based on the biomarkers to predict possible drug‐targeting signal pathways, hoping to yield novel insights, and promote the progress of the anticancer therapeutic strategy.

**Conclusion:**

We succefully constructed an autophagy‐related mRNA/miRNA/TF/Immune cells network based on a 10 state‐of art algorithm multi‐omics analysis, and screened the drug sensitivity dataset for detecting potential signal pathway which might be possible autophagy modulators' targets.

## Graphical abstract

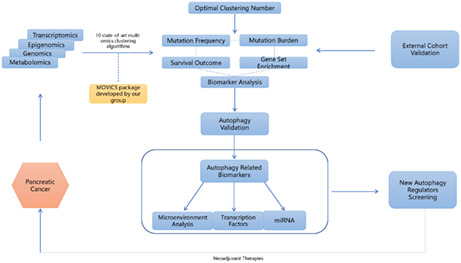

## 1 INTRODUCTION

Pancreatic cancer, which ranks the 7th leading cause of cancer‐related deaths all over the world, would probably surpass breast cancers by 2025 due to its poor prognosis.^1^, ^2^, ^3^ Tumorigenesis risk factors of the major type of pancreatic cancer, pancreatic ductal adenocarcinoma (PDAC), include intrinsic gene mutations (KRAS, TP53, SMAD4, and CDKN2A most commonly presented) and chronic inflammation caused by alcohol consumption, diabetes, and obesity.^3^, ^4^ Despite forced expression of mutation genes having been proven, there remain few therapeutically actionable‐targeted modalities due to its insidious advancement and crosslink between critical genetic drivers leading to escape. Thus, it is of great value to investigate novel biomarkers and further means by which PDAC can be therapeutically targeted.

Autophagy is a conserved degradation process through a lysosome‐dependent mechanism triggered by stress and may cause resistance to immune‐targeted therapies.^5^ It was proved to be highly upregulated in the later stages of PDAC and required for continued malignant progress compared with normal pancreatic duct cells,^6^ mediated by controlling the nuclear retention of microphthalmia transcription factor family members through ERK/MAPK2 pathway.^7^, ^8^ Increased autophagy sequestered MHC‐I on PDAC cells and weaken the antigen presentation and antitumor immune response, indicating the potential therapeutic strategies of autophagy inhibitors.^9^ However, there remain some controversies about autophagy regulating therapies in other tumors, along with the scarce data about autophagy‐related prognostic biomarkers and pharmacological modulation models, leaving the putative target exploration space in preclinical models.^10^ Although specific autophagy regulators recognized by the world scientific community are numbered (Chloroquine and hydroxychloroquine for inhibition; rapamycin [mTOR inhibitor] and temsirolimus for enhancement) and have indeterminate results in some trials combined with cytotoxic chemotherapy or immunotherapy, other targets related to autophagy and combination strategies have gotten the attention by pharmacists and doctors.^10^, ^11^

As a heterogeneous disease, traditional approaches fail to see the wood for the trees and lack robustness in identifying the classification and regulators modules. The advent of high‐throughput gene technologies, which provided multi‐omics data about genomics, transcriptomics, epigenomics, metabonomics, radiomics, and so on, enables clustering multi‐omics data in an integrated way to get different subgroups related to survival information, revealing the potential relationship between different omics and get further systems‐level insights.^12^, ^13^ Kwon, et al.^14^ included miRNA and mRNA data to conduct a comprehensive analysis by using support machine modeling (SVM) with leave‐one‐out cross‐validation (LOOCV), which identified hundreds of multi‐markers between PDAC and normal tissue. Deepa, et al.^15^ tried to generate a system‐level network of PDAC based on the omics data obtained from the rank‐based meta‐analysis (mRNA, miRNA, DNA methylation) in a multistep way, and have found eight potential hub genes related to survival: RASA1, IRS1, E2F3, HMGA2, ACTN1, NUAK1, SKI, and DLL1. Nguyue, et al.^16^ introduced the random forests (RF) model to validate the diagnostic value of multiplex biomarkers candidates in pancreatic cancer and improved the interpretability of multi‐omics mining. Nitish, et al.^17^ utilized a traditional statistic method of logistic regression to identify the genes related to PDAC patients' survival information. After that, researchers tried some different clustering methods and group numbers to divide PDAC into different subgroups. Autoencoder, iCluster, and IntNMF have been implemented respectively to enhance the robustness of the multi‐omics model,^18^, ^19^, ^20^ trying to decipher some subtle molecular characteristic models. Apart from these, Teresa, et al. successfully constructed a multi‐omics model by applying DIABLO integrating methods and compared transcriptional and mutational profiles between well‐differentiated neuroendocrine tumors and ductal adenocarcinomas.^21^ All of these just open the door to draft a new blueprint and showed the feasibility of this method to construct a robust and sensitive enough multi‐omics model, not only in differentiating the subtypes of PDAC, but also in some other pancreatic diseases such as early cystic lesion.^22^ However, all of them just utilized single statistic method or part of existing dataset, and no one have tried combined several thresholds of different omics biomarkers with the aiming of revealing the autophagy classification and drug sensitivity related to it.

So as discussed above, we constructed a PDAC multi‐omics classification model to reveal the autophagy classification by performing 10 state‐of‐the‐art multi‐omics clustering algorithms (ConsensusClustering, COCA, NEMO, PINSPlus, iClusterBayes, moCluster, SNF, LRA, CIMLR, and IntNMF), and then calculated the potential autophagy regulators hoping to spur the progress in this from‐zero‐to‐hero area.

## 2 MATERIAL AND METHODS

### 2.1 Multiple omics transcriptome datasets obtaining

TCGA‐PAAD (https://portal.gdc.cancer.gov/projects/TCGA‐PAAD, *n* = 185) was obtained as the training cohort for further analysis. The mRNA, lncRNA, miRNA, DNA methylation, somatic mutations, and clinical information of adenomas and adenocarcinomas subtype were enrolled. mRNA and lncRNA high‐throughput sequencing row count were downloaded by “TCGAbiolinks” R packages.^23^ Annotated to GENCODE27 file, former's gene symbols were transformed by matching the Ensembl IDs. And the latter were identified by Vega (http://vega.archive.ensembl.org/) and classified as noncoding, 3prime overlapping ncRNA, antisense RNA, lincRNA, sense intronic, sense overlapping, macro lncRNA, and bidirectional promoter lncRNA subtypes. For greater comparability between samples, we got the transcripts per kilobase million (TPM) by transforming the numbers of fragments per kilobase million (FPKM). When it comes to miRNA, downloading was performed through UCSC Xena (https://xenabrowser.net/) and translation was done via the R package “miRNAmeConverter” in miRbase version 21.0. And Xena database was screened for DNA methylation profile. Clinical information including progression‐free survival (PFS), overall survival (OS), and somatic mutations was obtained from cBioPortal database (https://www.cbioportal.org/).

### 2.2 External PADC transcriptome datasets obtaining

Totally five external transcriptome expression profiles (PACA‐AU; PACA‐CA; PAEN‐AU; GSE57495; GSE78229) were prepared for validation sets. Donors' information, specimen information, and expression sequence of PACA‐AU (*n* = 461), PACA‐CA (*n* = 317), and PAEN‐AU (*n* = 67) were downloaded from International Cancer Genome Consortium (ICGC, https://dcc.icgc.org/) and then cleaned for further analysis. GSE57495^24^ (sequenced by Rosetta/Merck Human RSTA Custom Affymetrix 2.0 microarray, *n* = 63) and GSE78229^25^ (sequenced by Affymetrix Human Gene 1.0 ST Array, *n* = 50) were downloaded from Gene expression omnibus (https://www.ncbi.nlm.nih.gov/geo/). Each gene symbol was transferred to multiple probe IDs in accordance with specific platforms' annotation file, and the cross‐dataset batch effect was cleaned by “sva” package.^26^

### 2.3 Calculating optimal number for clustering and integration of multi‐omics for subtypes visualization

The log2 transformation of transcriptome expression TPM were first finished. Probes at CpG islands of promotor region were gotten for methylation analysis, with the definition of median β value of those ≥1 mapping probes. When it comes to mutation matrix, mutant status was divided into two types: 1‐mutated (deletion/insertion, frameshift deletion/insertion, splice site or translation start site mutation, missense/nonsense/nonstop mutation) and 0‐wildtype. Univariate Cox proportional hazards regression worked as an efficient method to reduce data dimension for clustering analysis, and only those factors highly related to RFS were retained. To get an optimum number of clusters *k* at a low noise level but at the same retaining important information, two statistic parameters (clustering prediction index, CPI^27^ and Gaps‐statistics^28^) were calculated simultaneously. The sum of CPI and Gap‐statistics will be calculated and sorted for a rank. Higher rank of means bigger difference among molecular landscapes. Then, the K‐M analysis will be performed to decide the final clustering number. A 10 state‐of‐the‐art integrative calculation was performed based on the strategy of an unsupervised algorithm.^29^ To improve the clustering robustness, we did consensus ensembles for later integration of the clustering results derived from different algorithms and got pairwise similarities.

### 2.4 Comparing clustering outcomes and Genome‐wide signaling pathways

The overall nominal *p* value was calculated by log‐rank test. Pairwise comparison and derives adjusted *p* values were calculated if more than two subtypes are identified. And the Kaplan–Meier (KM) curves were created to illustrate the survival outcomes. Considering tumor‐specific genomic lesions and alterations that may affect immunotherapy, we calculated the total mutation burden (TMB) and fraction genome altered (FGA) among different subtypes. We chose edgeR and DESeq2 for RNA‐Seq count data and limma for microarray profile or normalized expression data to identify differentially expressed genes (DEGs). The most differentially expressed genes sorted by log2FoldChange were chosen as the biomarkers for each subtype. The R package “GSVA” was applied to calculate the single‐sample gene set enrichment analysis (ssGSEA) enrichment score and identify the subtype‐based functional pathways. Gene Ontology (GO, c5.bp.v7.1.symbols.gmt) from The Molecular Signatures Database (MSigDB, https://www.gsea‐msigdb.org/gsea/msigdb/index.jsp) and Kyoto Encyclopedia of Genes and Genomes (KEGG, https://www.genome.jp/kegg/) were carried out for reference.

### 2.5 External cohort validation

In this study, we performed two cross‐platform and cross‐species model‐free approaches for subtype similarity and reproducibility prediction in validation cohort: nearest template prediction (NTP),^30^ partition around medoids (PAM)+in‐group proportion (IGP) statistic.^31^, ^32^ What is more, we also created the KM curve to evaluate how consistent the different prediction results are based on clinical information.

### 2.6 Differentially expressed autophagy‐related genes (DEARGs) identification and Protein–Protein Interaction Networks (PPI) construction

All the 232 genes directly or indirectly related to autophagy as described in literature were collected from Human Autophagy Database (HADb, http://www.autophagy.lu/index.html) and intersected with all the DEGs upregulated in poor prognosis group called as DEARGs. The computational prediction of physical and functional interactions among proteins was conducted by The Search Tool for the Retrieval of Interacting Genes (STRING database, V11.0).^33^ Topological characteristic visualization and hub genes revelation were finished by CytohHubba plugin of Cytoscape.^34^

### 2.7 Microenvironment infiltrated cells and correlation analysis

Immune cell‐related gene signature was analyzed by the Cell Type Identification by Estimating Relative Subsets of RNA Transcripts (CIBERSORT)^35^ for calculating the fraction of 22 immune cell subpopulations. To identify the immune infiltration difference among groups, a Wilcoxon rank‐sum test was performed. For further information about the relationship between DEARGs and infiltrated immune cells, the Pearson correlation was applied.

### 2.8 Prediction of transcription factors (TFs) and miRNA that regulate DEARGs

Enrichr, as an updated curated gene resource and search engine, enables us to predict the TFs, mRNA, and miRNA regulating network. TRANSFAC, JASPAR, and miRTarBase dataset were included for further biological discoveries.

### 2.9 Drug sensitivity and immune therapies response analyses

Paul, et al. first developed a coupling method comparing the in vitro and in vivo gene expression to predict the response to specific drug of cancer patients.^36^ Borrowing this idea, GDSC (https://www.cancerrxgene.org/) provided drug sensitivity and phenotype data information of 727 human cancer cell lines which were collected for our drug sensitivity analysis. All the predictions were finished by R package “pRRophetic”. Ridge regression was the efficient way to evaluate the half maximal inhibitory concentration (IC50) of potential autophagy‐related chemotherapeutic agent (accuracy prediction from the 10‐fold cross‐validation).

### 2.10 Statistics

All analyses were conducted by R4.1.0. Kaplan–Meier curves were depicted based on a log‐rank test. Hazard ratios (HR) and 95% confidence interval (CI) were estimated by Cox proportional hazards regression model. We analyzed continuous data by two‐sample Mann–Whitney test and categorical data by Fisher's exact test. We successfully constructed an R package “MOVICS” and embedded all above analytic processes in it.^27^ Statistically significance was defined as the two‐tailed *p* < 0.05. For evaluating the similarity and reproducibility of the acquired subtypes between discovery and validation cohorts, NTP and PAM+IGP statistic were carried out to calculate the Kappa index.

## 3 RESULTS

### 3.1 Multi‐omics integrative analysis and subgroups characteristics

**FIGURE 1:**
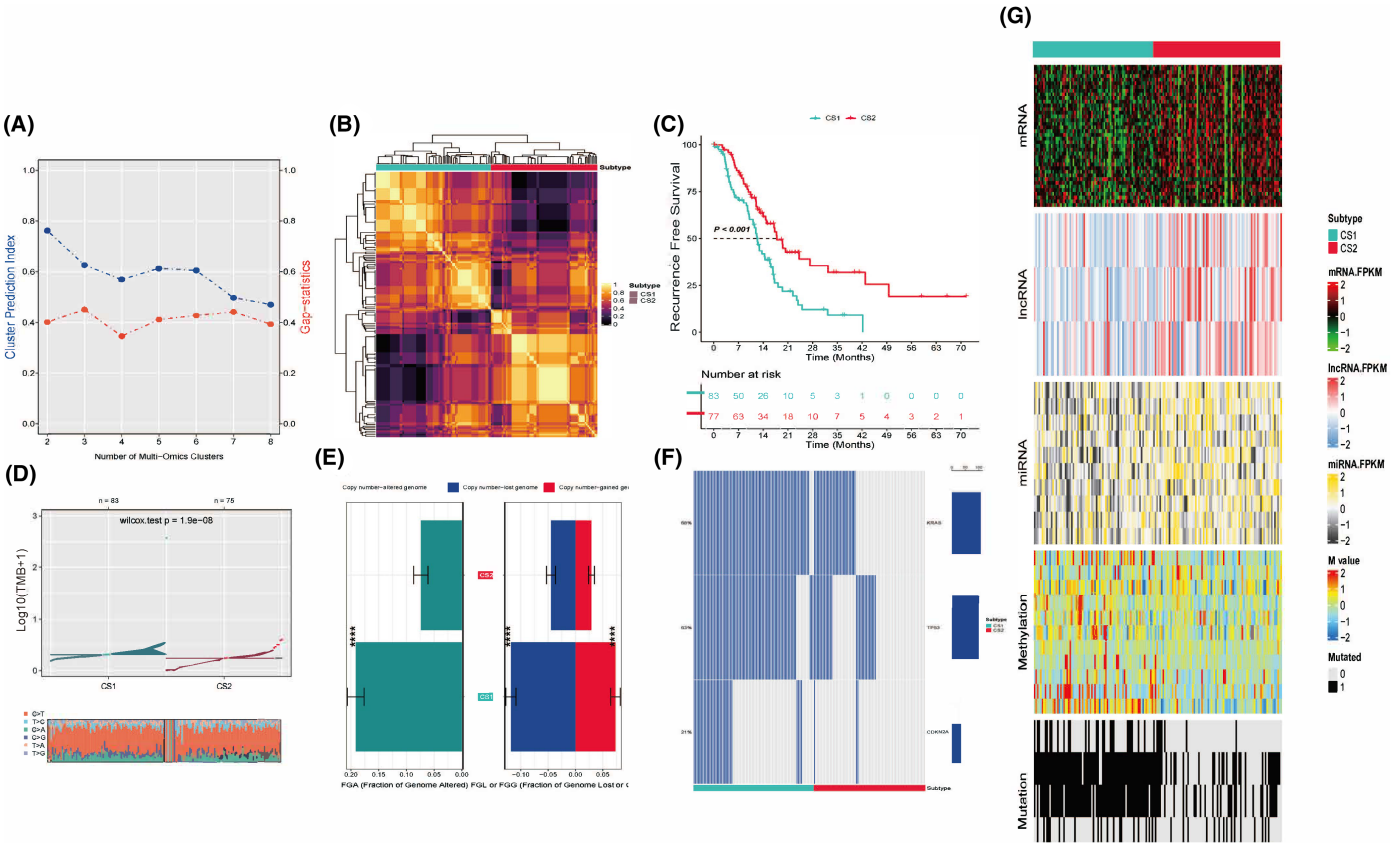
Integrated analysis and subgroup characteristics based on multi‐omics data. (A) CPI and gap‐statistic calculation revealed the optimal cluster number might between 2 and 4. The sum of CPI and Gap‐statistics will be calculated and sorted for a rank. Higher rank of means bigger difference among molecular landscapes. (B) Visualization of the 10 multi‐omics integrative clustering algorithms results with cluster number of 2. After get all results from specified algorithms, MOVICS calculates a consensus matrix CM = ∑(*t*_max_, *t* = 1)M^(*t*)^, and cm_ij_∈[0,10]. Such matrix was represented by clustered purple and gold colors in a stylish way to show a robust pairwise similarities for samples because it considers different multi‐omics integrative clustering algorithms. (C) KM curve reveals the statistical significance between two subgroups' clinical outcomes. Significant better prognosis of group 2 indicates the legitimacy of setting cluster number as 2 (*p* < 0.001). (D) Total mutation burden (TMB) is higher in CS1, and the major one of transition is C>T. (E) FGA and specific gain (FGG) or loss (FGL) per sample are more common in CS1. (F) KRAS, TP53, and SMAD4 are the first three mutation detected. (G) Comprehensive heatmap based on consensus across 10 algorithms about mRNA, lncRNA, miRNA, methylation, and mutation condition

After getting the elites data from mRNA, miRNA, lncRNA, methylation, and mutation profiles by univariate Cox proportional hazards regression or freq‐method for binary data, 160 patients' data with reduced dimension for clustering analysis remained. Information of mRNA, miRNA, lncRNA, and methylation features got for clustering could be found in Tables S1–S4. Just as what has been shown in Figure 1A, the optimal subgroup cluster number indicated by CPI and Gaps‐statistics is 2. To be more cautious, we have also tested other numbers of clusters which have been reported more than 2 or when the two methods scores were more approximate. But when the cluster number came larger than 2, some subgroup might contain such a small part of patients that unable to continue the analysis, or some subgroups' survival outcomes were too similar to be differentiated, reducing the need for a larger clustering number (Figures S1 and S2). So, integrated 10 multi‐omics analyses using iClusterBayes, moCluster, CIMLR, IntNMF, ConsensusClustering, COCA, NEMO, PINSPlus, SNF, and LRA were performed to divided the PADC patients into two subgroups (CS1, *n* = 83 and CS2, *n* = 77) for robustly distinctive molecular patterns (Figure 1B). As Figure 1C, clinical outcomes showed notable difference in KM curve: CS2 had a longer recurrence‐free survival time versus CS1 (*p* < 0.001).

After identification of subtypes, other characteristics of each subtype from multiple aspects should be explored for downstream analyses. In the larger scheme, TMB (Figure 1D) and fraction genome altered (FGA, Figure 1E) in CS1 genome are much higher than them in CS2 (*p* < 0.001), and CS1 presented a landscape of lower mRNA, lncRNA, and miRNA expression along with higher methylation, chromosomal instability, and mutation rates (Figure 1G). All of these indicated the relationship between higher aberration and poor prognosis, in accord with our cognition. To be more specific, MIR3142HG, AC022182.1, and AL358472.2 were the three lncRNAs mostly related to survival outcomes between two groups (*p* < 0.001), and hsa‐miR‐98‐5p, hsa‐miR‐218‐5p, hsa‐miR‐140‐5p, hsa‐miR‐146a‐5p, hsa‐miR‐29c‐5p, hsa‐miR‐653‐5p, hsa‐miR‐3613‐5p, hsa‐miR‐145‐3p, hsa‐miR‐374a‐3p, hsa‐miR‐590‐3p were found to be the first 10 miRNAs presenting high discriminative ability (*p* < 0.001). What is more, cg00803804, cg00888162, cg01971137, cg06000963, cg11174851, cg13361843, cg18701590, cg24950336, cg25087487, and cg26546557 were the sites appeared most often in methylation (*p* < 0.001). And single primary signature of C>T transitions at CpG sites was the dominant one. Besides these, CS1 harbored significantly more mutations of KRAS (*p* adj <0.001), TP53 (*p* adj <0.001), SMAD4 (*p* adj <0.001), TTN (*p* adj <0.001), and CDKN2A (*p* adj <0.001) (Figure 1F; Table 1).

**TABLE 1:** Independent test between subtype and mutation

Gene (mutated)	TMB	CS1	CS2	*p* value	*p* adj
KRAS	109 (68)	80 (96.4)	29 (37.7)	6.45e‐17	3.23e‐16
TP53	101 (63)	74 (89.2)	27 (35.1)	8.47e‐13	2.12e‐12
SMAD4	36 (22)	21 (25.3)	15 (19.5)	4.50e‐01	4.50e‐01
TTN	27 (17)	17 (20.5)	10 (13.0)	2.91e‐01	3.64e‐01
CDKN2A	34 (21)	31 (37.3)	3 (3.9)	1.31e‐07	2.18e‐07

*Note*. Values in parentheses are percentages.

### 3.2 Biomarker identification and external datasets validation

**FIGURE 2:**
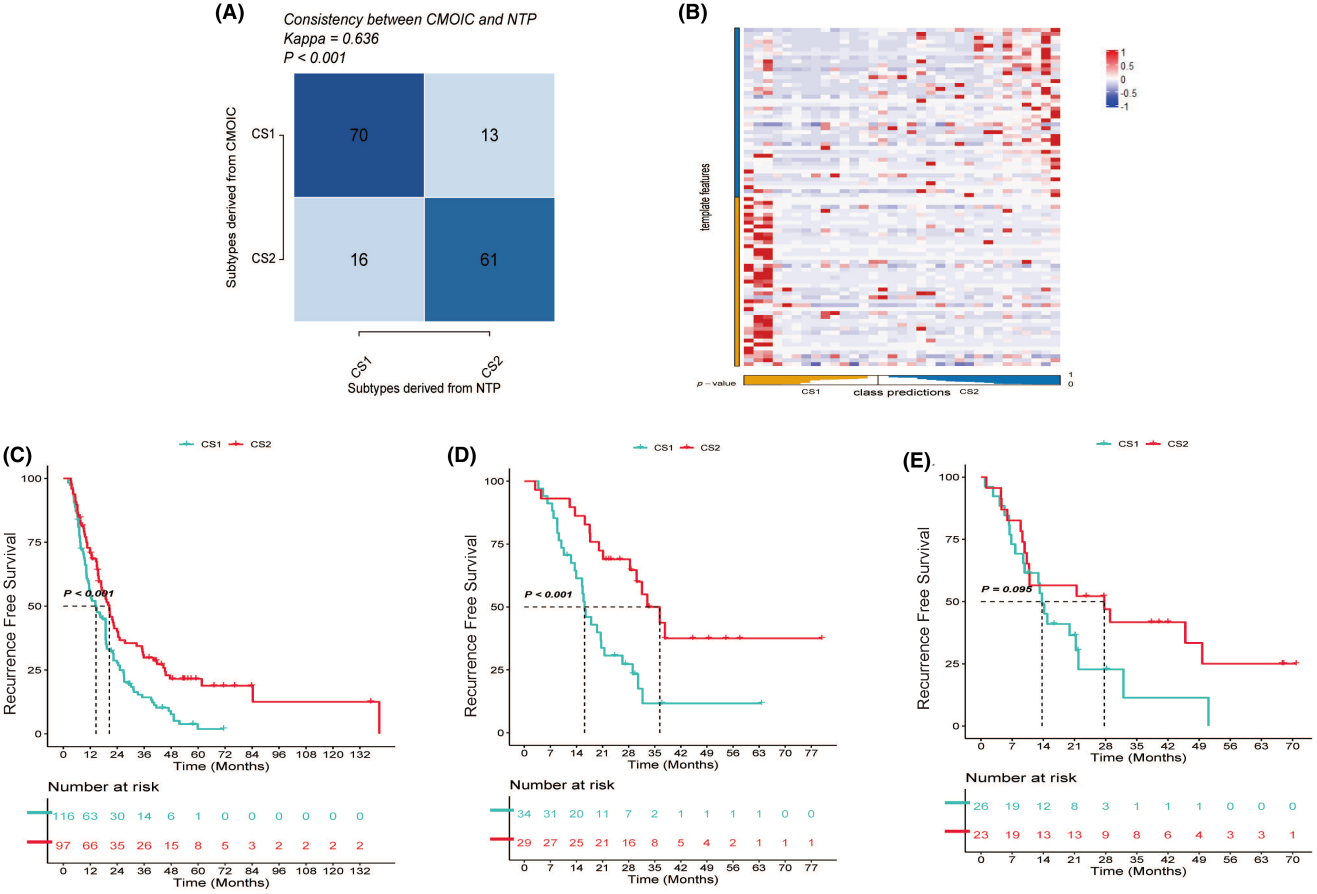
External validation. (A) Kappa value of PACA‐AU (Kappa = 0.636, *p* < 0.001) showed high similarity between NTP and CMOIC (CS1 vs. CS1 and CS2 vs. CS2). (B) PAM heatmap of PAEN‐AU revealed the similar structure allocation between training outcomes and external validation. For both CS1 or CS2 in template features and class predictions, higher PAM (red dots) could be observed to cluster. (C) KM curve reveals the statistical significance between two subgroups' clinical outcomes in PACA‐CA. Significant better prognosis of group 2 indicates the legitimacy of setting cluster number as 2 (*p* < 0.001). (D) KM curve reveals the statistical significance between two subgroups' clinical outcomes in GSE57495. Significant better prognosis of group 2 indicates the legitimacy of setting cluster number as 2 (*p* < 0.001). (E) KM curve reveals the statistical significance between two subgroups' clinical outcomes in GSE78229. Significant better prognosis of group 2 indicates the legitimacy of setting cluster number as 2 (*p* = 0.095)

Potential predictive biomarkers pass the significance threshold (*p* adj value <0.05) were detected as differentially expressed genes (DEGs) by limma method. These biomarkers not overlap with any biomarkers identified for other subtypes have been shown in Figure S3 as upregulated one and Figure S4 as downregulated one. Then, these biomarkers were used to run nearest template prediction in external cohorts for validity and accuracy testing. To deal with the multi‐classification problem cross different omics data, we applied two model‐free subtype prediction approaches (NTP and PAM) along with survival outcomes validation. For PACA‐AU, the Kappa value was 0.636 (*p* < 0.001), indicating the high consistency between NTP and consensus analysis outcome (deep blue color blocks in Figure 2A). For PAEN‐AU, red blocked could be observed to enrich in quadrants I and III the heatmap of cross‐platform NTP (*p* < 0.001), showing the similar prediction structure between known biomarkers and external validation (Figure 2B). Survival outcomes similarities could also be verified in external datasets: for PACA‐CA (Figure 2C, *p* < 0.001), GSE57495 (Figure 2D, *p* < 0.001), and GSE78229 (Figure 2E, *p* = 0.095), the separate KM curves had become notably visible, revealing similar clinical outcomes among different datasets that CS1 patients have poor prognosis. Especially, GSE78229 might not be so much statistically significant compared to other datasets (*p* = 0.095) possibly due to a smaller sample size, but the obvious separation of two groups' curves could be seen over time, which left our reasons to draw a conclusion that the prognosis of CS2 was better than CS1 in GSE78229.

### 3.3 Construction of PPI and TF/mRNA/miRNA regulatory network

**FIGURE 3:**
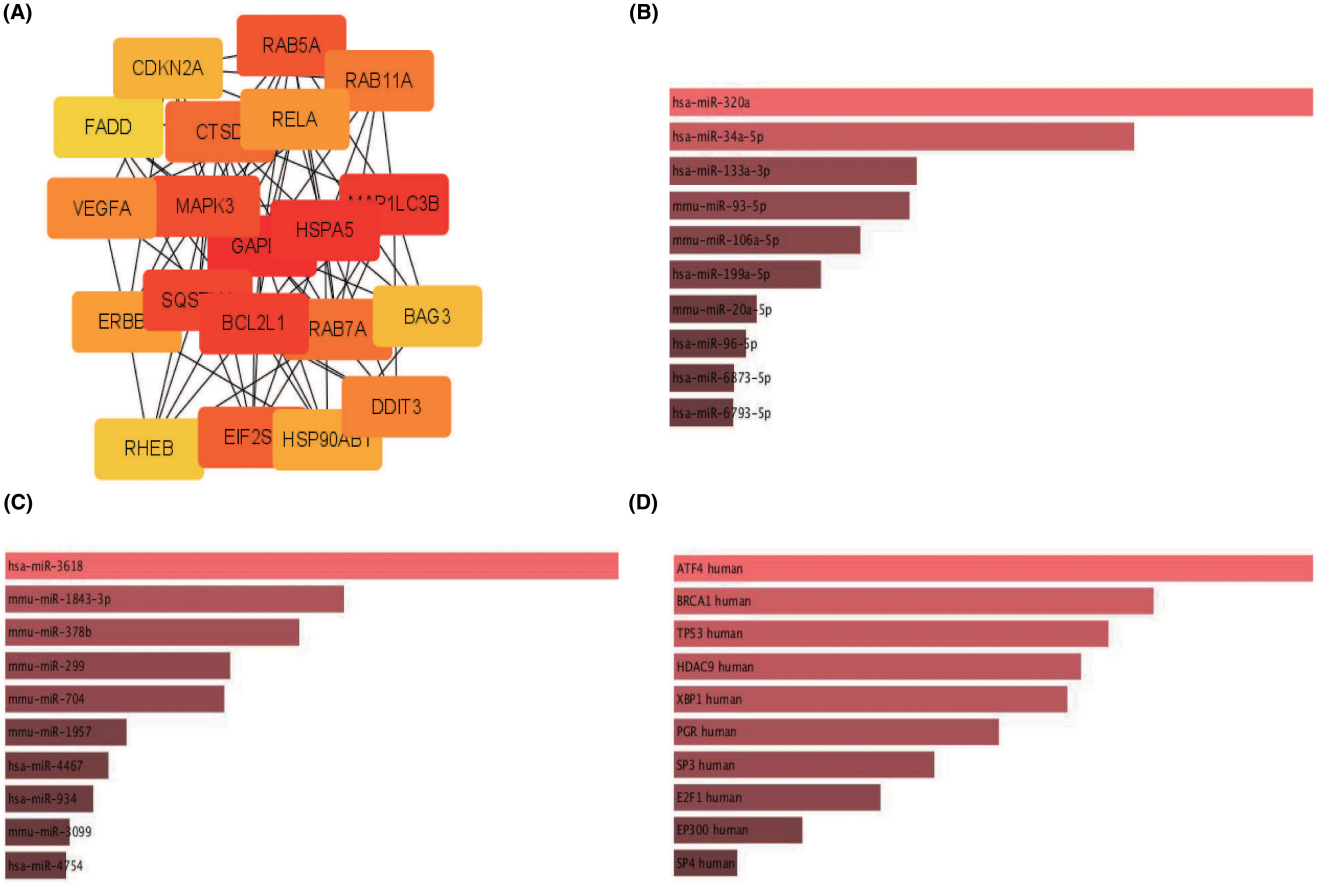
PPI network and mRNA/miRNA/TF interaction construction. (A) Top 20 significant hub genes related to autophagy and PDAC. Hub genes were obtained through CytoHubba plugin (MCC algorithems), and deeper red means higher Hubba nodes score. (B) Possible miRNA regulators predicted by miRTarBase. Shallower red means smaller *p* adj value and longer length represents higher combined *Z* score. (C) Possible miRNA regulators predicted by TargetScan. Shallower red means smaller *p* adj value and longer length represents higher combined *Z* score. (D) Possible TF regulators predicted by TRANSFAC and JASPAR PWMs. Shallower red means smaller *p* adj value and longer length represents higher combined *Z* score

We collected 232 genes directly or indirectly related to autophagy from HADb and intersected with the DEGs as DEARGs. DEARGs upregulated in CS1 subgroup were extracted for further analysis, and the top 20 highest degree hub nodes were selected to construct the PPI network (Figure 3A). GAPDH, MAPK3, RHEB, SQSTM1, EIF2S1, RAB5A, CTSD, MAP1LC3B, RAB7A, RAB11A, FADD, CFKN2A, HSP90AB1, VEGFA, RELA, DDIT3, HSPA5, BCL2L1, BAG3, and ERBB2 were the 20 hub genes related to autophagy and PDAC.

To deeper the exploration of mRNA/TF/miRNA regulation mechanism, some other databases were applied: miRTarBase provided the possible miRNA regulator such as hsa‐miR‐320a, hsa‐miR‐34a‐5p, and has‐miR‐133a‐3p; TargetScan provided some other potential regulator such as has‐miR‐3618, mmu‐miR‐1843‐3p, and mmu‐miR‐378b; TRANSFAC and JASPAR PWMs suggested some possible TF might be interacted with mRNA, such as ATF4, BRCA1, TP53, HDAC9, and XBP1.

### 3.4 Microenvironment infiltrated cells and correlation analysis between autophagy‐related genes and immune cells

**FIGURE 4:**
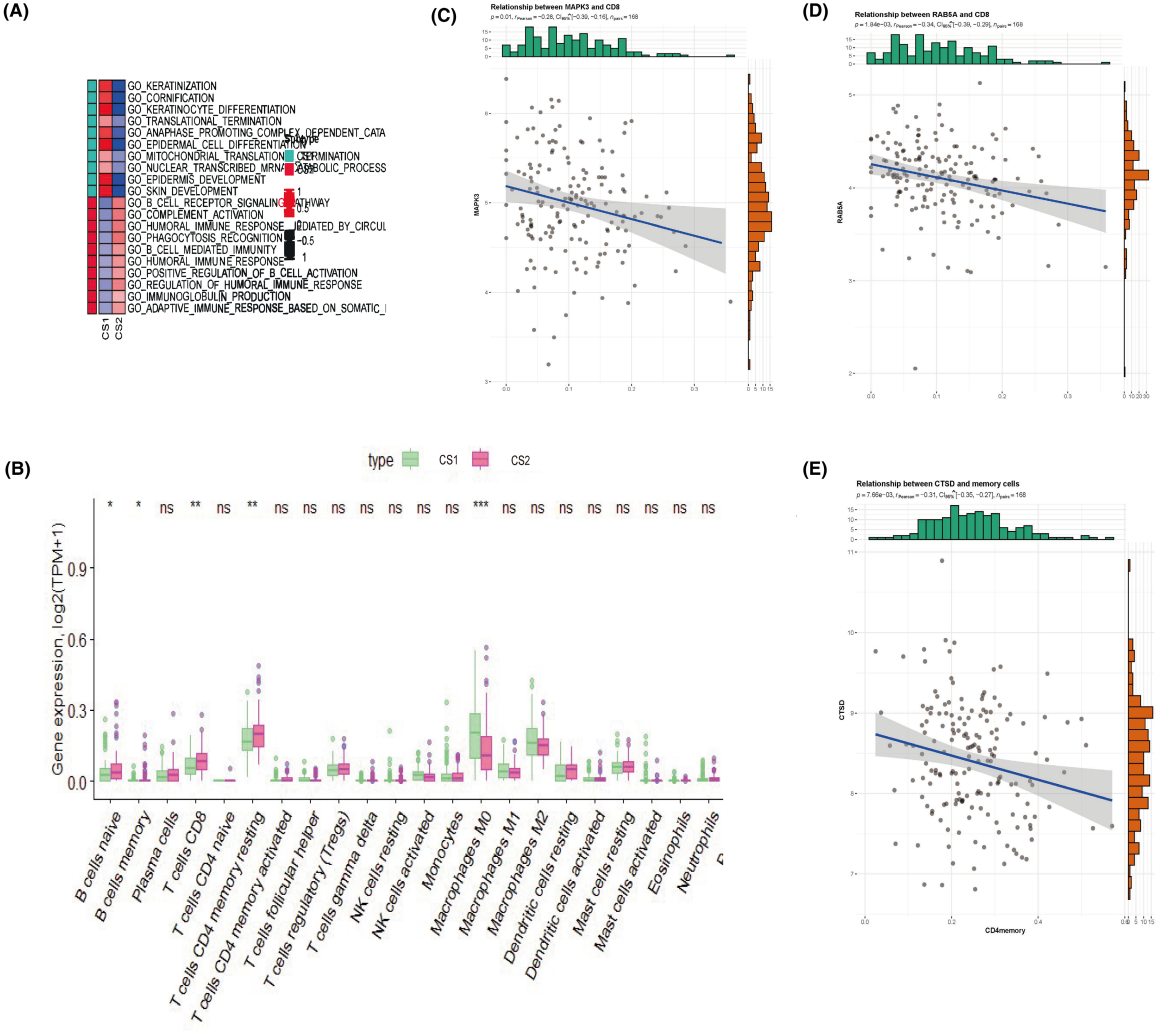
Functional pathway enrichment and immune‐related analysis. (A) GO biological processes gene sets enrichment analysis revealed higher immune activity in CS2 and higher epidermic keratinization activity in CS1. In the first column, green blocks refer to molecular function in GO analysis, and red blocks refer to biological process in Go analysis. In the second and third columns, red blocks refer to upregulation and blue blocks refer to downregulation. (B) Infiltrated immune cells landscape of CIBERSORT indicates the significant different infiltration density of B cell naive, B cell memory, T cells CD8, and Macrophage M0 between CS1 and CS2. (C) Pearson correlation plot between MAPK3 and CD8^+^ cells (*p* = 0.01, *r* = −0.28). (D) Pearson correlation plot between RAb5a and CD8^+^ cells (*p* < 0.001, *r* = −0.34). (E) Pearson correlation plot between CTSD and CD4^+^ memory cells (*p* < 0.001, *r* = −0.31)

Subtype‐specific functional pathways based on DEA were calculated by GSEA in Figure 4A based on GO biological processes from MSigDB. It is obviously that the CS2 subgroup, which has a better prognosis, showing increased immune activation related to adaptive immune reaction, humoral immune response, B‐cell regulation, and complement activation. In contrast, CS1 subgroup with grave prognosis presented to be enriched in epidermis keratinization, development, differentiation, and transformation. The enrichment condition seemed to imply the immune activities difference between two subgroups, so infiltrated immunocytes were analyzed for revealing the possible different tumor microenvironments (Figure 4B). Compared to CS1, there exist more immunologic effector cells such as naive B cells (*p* < 0.05), memory B cells (*p* < 0.05), CD8^+^ T cells (*p* < 0.01), memory resting CD4^+^ T cells (*p* < 0.01) but less M0 macrophages (*p* < 0.001) in CS2, which may indicate the stronger immune toxicity against PDAC in CS2. Considering the unclear condition between PDAC autophagy‐related genes expression and infiltrated immune cells, we conducted a Pearson correlation analysis to underlying the possible interaction of them (Table S5). The upregulated autophagy‐related genes showed extensive positive interaction with each other, such as SQSTM1 and RELA, FADD and RELA, ERBB2 and FADD, MAP1LC3B and RAB51, MAP1LC3B and RAB7A, MAP1LC3B and DDIT3. What is more, the upregulation of autophagy genes seemed to be related to the activation of immune effector cells, such as MAPK3 and CD8^+^ T cells (Figure 4C, *p* = 0.01, *r* = −0.28), RAB5A and CD8^+^ T cells (Figure 4D, *p* < 0.001, *r* = −0.34), CTSD and memory CD4^+^ T cells (Figure 4E, *p* < 0.001, *r* = −0.31). Besides these, negative correlation could also be observed between different immune cells, such as M0 macrophages and CD8^+^ T cells, M0 macrophages and resting CD4^+^ T cells, naive B cells, and CD8^+^ T cells.

### 3.5 Therapeutic response analyses

**FIGURE 5:**
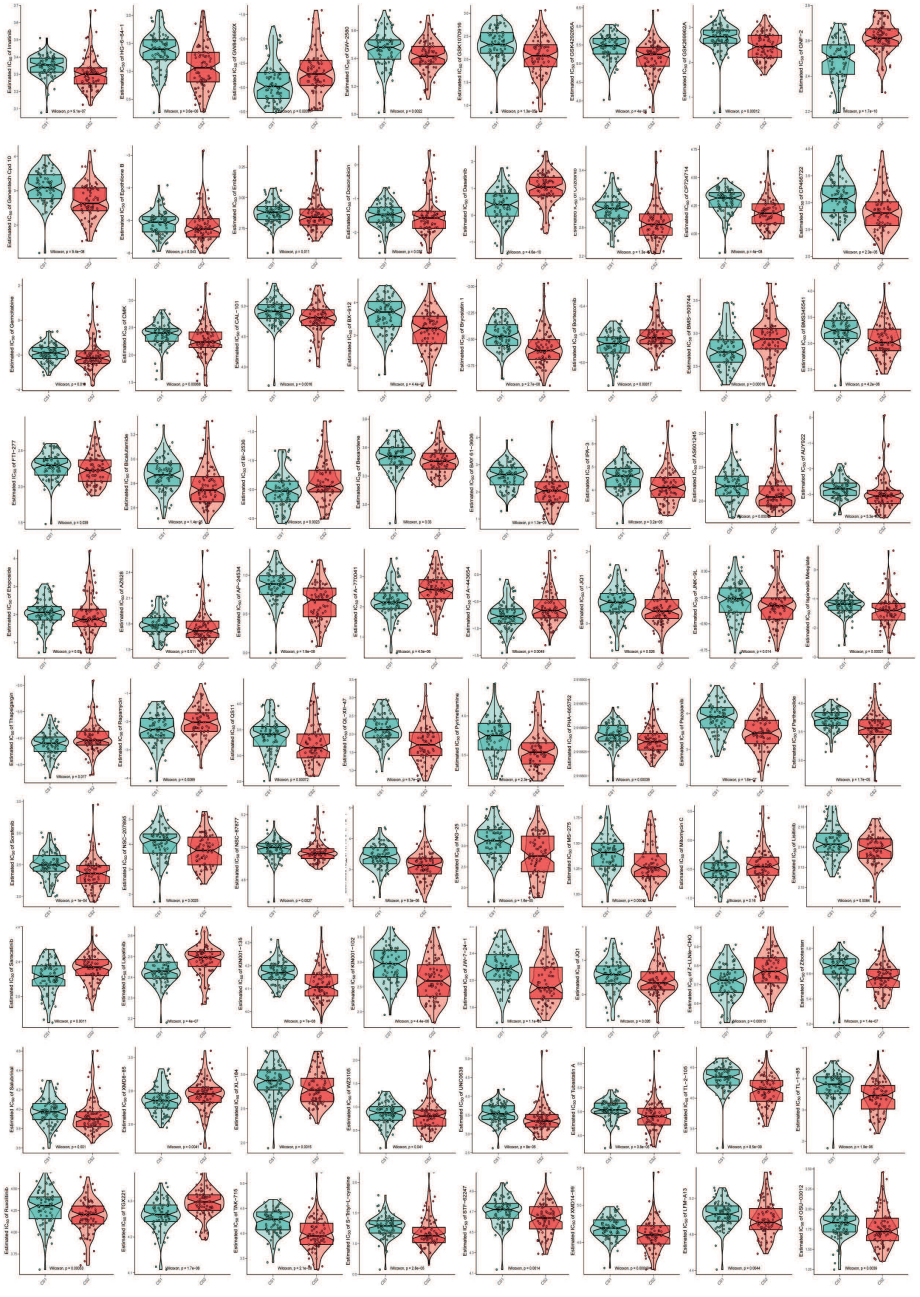
Boxviolins for estimated IC_50_ of different drugs between two PDAC subtypes. All drugs whose IC_50_ are significantly different between two groups are presented on Figure 5: imatinib, HG‐6‐64‐1, GW843682X, GW2580, GSK1070916, GSK429286A, GSK269962A, GNF‐2, Cpd10, EpothiloneB, embelin, doxorubicin, dasatinib, crizonib, CP724714, CP466722, GEMCITABINE, cmk, cal‐101, bx912, Bryostatin1, bortezomib, BMS‐509744, BMS345541, FTI277, bicalutamide, BI2536, bexarotene, BAY61‐3606, IPA3, as601245, AUY922, Etoposide, AZ628, AP24534, A770041, A443654, JQ1, JNK‐9 L, mesylate, thapsigargin, rapamycin, QS11, QL‐XII‐47, pyrimethamine, PHA665752, pazopanib, parthenolide, sorafenib, NSC207895, nsc87877, NG25, MS275, mitomycin c, listinib, saracatinib, lapatinib, KIN001135, KIN001102, JW7241, JQ1, Zibotentan, ZLLNLECHO, Salubrinal, XMD885, XL184, WZ3105, UNC0638, Tubastatin A, TL2015, Tl185, Ruxolitinib, TGX221, TAK715, S‐trityl‐l‐cysteine, STF‐62247, XMD14‐99, LFM‐A13, OSU‐03012. Detail information for their IC_50_ and *p* value referred to Table S6

We screened the database with the R package “pRRophetic” for prediction of potential clinical chemotherapeutic responses related to our classification outcomes and autophagy genes expression condition (Figure 5; Table S6). Some drugs whose mechanism are related to autophagy‐induced tumorigenesis and drug resistance were included for analysis.

First, we compared some drugs' sensitivities whose targets had been proven to be related to autophagy, and the Autophagy—animal—Reference pathway could be found on KEGG (https://www.genome.jp/pathway/map04140). Rapamycin, working as a mTOR inhibitor to induce autophagy by targeting mTORC1, has a lower IC50 in CS1 (*p* = 0.0089). The mTOR is an effector and could be upregulated by PI3K/AKT/PKD and MAPK/Erk1/2 signaling pathways. TGX‐221 is more sensitive in CS1 against different PI3K isoforms (*p* < 0.001). And KIN001–102 (AKT inhibitor, *p* < 0.001), BX‐912 (PKD1 inhibitor, *p* < 0.001), and OSU‐03012 (AKD and PKD inhibitor, *p* = 0.0039) are more sensitive in CS2, while A‐443654 is more sensitive in CS1 (AKD inhibitor, *p* = 0.0049). For Ras/Raf/Mek1/2/Erk1/2, the other upstream positive signaling pathway of mTOR, some drugs were included for comparison: Ras and Raf inhibitor AUY922 has lower IC50 in CS2 (*p* < 0.001); Raf inhibitor HG‐6‐64‐1 (*p* < 0.001), Sorafenib (*p* < 0.001), and AZ628 (*p* = 0.011) are sensitive to CS2.

Next, potential new signal pathway targets were included for prediction based on our multi‐omics regulation network. P53 activator NSC‐207895 which inhibits mTOR, has a lower IC50 in CS2 (*p* = 0.0025). Bryostatin 1, a macrocyclic lactone inhibits the cell‐signaling enzyme protein kinase C (PKC), has a lower IC50 in CS2 (*p* < 0.001). The sensitivity of two subgroups to some autophagy regulators were also compared in our analysis profile. The autophagy inhibitor Thapsigargin is more sensitive in CS1 (*p* = 0.017), and autophagy activators Etoposide (*p* = 0.05) and doxorubicin (*p* = 0.037) are more sensitive in CS2.

Anything else, some other drugs showed different sensitivity between two subgroups have been detected, although the relationship between drug mechanism and autophagy is unclear, such as Jak inhibitors, Syk inhibitors, IKK inhibitors, ITK inhibitors, Rock inhibitors, c‐Met inhibitors, p53/MAPK inhibitors, and so on (detail information in Figure 5).

## 4 DISCUSSION

Considering the refractory to most treatments and metastasis, PDAC leaves us a research priority to uncover the mechanism of tumor heterogeneity and search for effective therapies. For defining and refining the subtypes of PDAC based on different expression patterns to improve personalized treatments, several studies have implemented some single integrative methods to analyze the current omics data. In this study, we leveraged 10‐state‐of‐the‐art multi‐omics clustering algorithms (ConsensusClustering, COCA, NEMO, PINSPlus, iClusterBayes, moCluster, SNF, LRA, CIMLR, and IntNMF) for constructing a PDAC multi‐omics classification model, including mRNA, lncRNA, miRNA, methylation, and mutation datasets. We not only stratified the subtypes and identified the biomarkers in a more accurate and roust way, but also revealed the autophagy‐related genes and proteins landscape from the multi‐omics consensus outcome for the first time. What is more, we screened the GDSC database and created a comprehensive drug sensitivity analysis spectrum based on prediction network we built to reveal the potential autophagy regulators.

Taking clustering numbers in previous studies and the survival analysis outcomes in our studies based on two statistical functions, the optimal number was finally defined as 2 to reduce noise and intergroup similarity, in accordance with the pathology classification of PDAC (basal‐like/squamous and classical/pancreatic progenitor). To make sure the multi‐omics integrated analysis containing different statistical methods and expression profiles have a stronger robustness, external validation was performed in five datasets, showing the potential of our comprehensive analysis to elucidate the subtle underlying mechanism from different subgroups.

The most notable findings of MOVICS in PDAC were similar to some previous outcomes: patients in CS1 suffered from unfavorable prognosis tended to have genomic instability (higher mutation burden and copy number altered genome) and higher epidermal development and translation. Recurrent mutations of KRAS, TP53, SMAD4, TTN, and CDKN2A which have been proven to be related to prognosis and drug sensitivity in many whole exome, whole genome and multi‐omics studies were unsurprisingly detected in our outcome in a more robust way.

Considering the difficulty in finding drugs directly targets those mutations or potential biomarkers, we changed our mind and focused on autophagy regulation therapy, trying to yield novel insights into the potential effective target of this disease. Highly activated autophagy or some autophagy‐related genes were detected to be related to poorer prognosis^11^ compared with normal tissue. We focused on the autophagy‐related genes expression and modification between different PDAC subgroups based on multi‐omics data. And we found that more DEARGs were upregulated in CS1, along with the poor prognosis. The top 20 hub autophagy‐related genes (GAPDH, MAPK3, RHEB, SQSTM1, EIF2S1, RAB5A, CTSD, MAP1LC3B, RAB7A, RAB11A, FADD, CFKN2A, HSP90AB1, VEGFA, RELA, DDIT3, HSPA5, BCL2L1, BAG3, and ERBB2) upregulated in CS1 might be the potential biomarkers or targets. MAP1LC3B, as a subfamily of MAP1LC3 (ATG8 protein), could be converted to a widely used autophagic flux indication marker during the extension phage of autophagy.^37^ It can be upregulated along with the poor prognosis by USP22 (ubiquitin‐specific peptidase 22) expression through MAPK1 pathway.^38^ Combination therapies targeted MAP1LC3 that affect autophagy in PDAC have been noticed in some researches: Combining gemcitabine and ionizing radiation would upregulate autophagy by increasing MAP1LC3 and BECN1 to suppress PDAC growth,^39^ while combing seaweed polyphenols and fractionated irradiation could increase the radiosensitivity of PDAC by inhibiting MAP1LC and autophagy the other way around.^40^ RAB5A, RAB7A, and RAB11A are small GTPase members from RAS oncogene family, having key roles in autophagosome formation and maturation.^41^ The mutations of KRAS could be observed in over 90% of PDAC, and Yihua Wang, et al. have demonstrated that inhibiting autophagy in RAS‐mutated cells could increase epithelial‐mesenchymal transition (EMT) and invasion by targeting the NF‐κB pathway via accumulation of SQSTM1/p62, indicating the potential therapeutic effect of NF‐κB inhibitors+autophagy inhibitors.^42^ Weifeng Liu, et al. also reported that parthenolide could induce apoptosis through autophagy by upregulating p62/SQSTM1, LC3II, and Beclin 1 in Panc‐1 cells.^43^ RHEB, a GTP‐bound protein, could activate mTORC1 to integrate mTOR and GTPase in autophagy process.^44^, ^45^ The crosstalk between phosphorylation of translational control (EIF2S1), inhibition of pro‐inflammatory signaling (STAT3), and upregulated autophagy was proved by Niso‐Santano M, et al.^46^

To establish a more comprehensive regulating network for potential autophagy regulators exploration, TRANSFAC, JASPAR, and miRTarBase dataset were included for miRNA and TF prediction. We provided further potential miRNAs (hsa‐miR‐320a, hsa‐miR‐34a‐5p, has‐miR‐133a‐3p, has‐miR‐3618, mmu‐miR‐1843‐3p, and mmu‐miR‐378b) might regulate those DEARGs. The relationship and mechanism of all these miRNAs and PDAC autophagy are waiting to be explored by lab work, indicating some new possible directions of PDAC research.

The top five TFs were predicted as ATF4, BRCA1, TP53, HDAC9, and XBP1. TP53 showed higher mutation rates in CS1 related to higher autophagy and poorer prognosis. And the regulation of TP53 on autophagy is double directions.^47^ Histone deacetylase (HDAC) 9 was found to suppress the autophagy in hypoxia condition such as ischemia/reperfusion.^48^, ^49^ And the performance of HDAC inhibitors have statistical significance between two subgroups in our analysis.

To get the immune landscape of PDAC, infiltrated immune cells and the relationship between DEARGs and immune cells were analyzed. Pharmacologically or genetically upregulate autophagy will increase the degrading of MHC‐I on cell surface, impeding the antigen presentation, and promoting immune escape.^9^ CS2 group tended to have a lower expression level of autophagy‐related genes and a higher immune‐activated level in our analysis, in accordance with previous conclusion. Pearson correlation analysis also showed a negative relationship between DEARGs and immune cells and a positive relationship among different DEARGs. The multilevel and complex regulation crosslink among different genes and cells should be validated in lab work.

Although inhibiting autophagy to sustain the MHC‐I expression on cell surface or stimulating autophagy to induce cell death both seem a logical step, there still remains few autophagy regulators going to the clinical trials. Chloroquine and hydroxychloroquine inhibiting autophagy and lysosomal functions will not work on its own and neither will things like combination with gemcitabine.^50^, ^51^, ^52^, ^53^ Meanwhile, rapalogue (derivative of rapamycin) showed potential to be an effective autophagy modulator by synergistic cytotoxic effect with mTOR inhibitors in some preclinical studies. But this PI3K‐AKT inhibitor finally failed to shown significant in PDAC possibly due to feedback loop escape.^54^, ^55^, ^56^, ^57^ Searching for isoform‐specific targeting drugs to modulate autophagy has caught researchers' attention for some years, such as PI3K inhibitors, ATG7 inhibitors, ATG1 (ULK) inhibitors, VSP34 inhibitors, and so on. But most of them failed to progress to further clinical assessment subjected to the controversy effects in tumor modulation, leaving us a research priority for novel possible drugs.

Based on the known autophagy‐related regulating network from KEGG or other previous studies, DEARGs and TFs, all the inhibitors and stimulators have crosstalk with autophagy in dataset were included for drug sensitivity calculation. Known PDAC autophagy‐related signal targets were included and some compounds showed different sensitivity between two subgroups: mTOR (rapamycin), PI3K/AKT/PKD (TGX‐221, KIN001‐102, BX‐912, OSU‐03012, and A‐443654) and Ras/Raf/Mek1/2/Erk1/2 (AUY922, HG‐6‐64‐1, Sorafenib, and AZ628). We also proposed and tested some novel possible targets, hoping to find effective autophagy regulation compounds, such as P38/MAPK (TAK‐715 and KIN001‐135), MEK5/Erk5 (XMD8‐85), JAK/STAT (AS601245 and Ruxolitinib), EG5 (S‐Trityl‐l‐cysteine), HDAC (MS‐275, Tubastatin A and Parthenolide), C‐MET (PHA‐665752), VEGFR (pazopanib), BTK (LFM‐A13), ERBB2 (CP724714, Lapatinib), ROCK (GSK269962A and GSK429286A), IKK (bms345541), ITK (BMS509744), eIF2 (Salubrinal), and so on.

There remain some limitations have to be admitted: First, all the calculation and prediction were based on the current data, and there may remain some other unknown mechanism have effects on autophagy to make uncertainties or controversies. It is a hard and costly task for our group to validate all the possible drugs, their effects on autophagy‐related pathways maps and therapeutic benefits in such a short time, especially during the period of zero‐COVID policy in China now. So, the exact effect and mechanism how these biomarkers and drugs regulate autophagy should be tested and explored in lab and clinical work. We believe our map will show directions to researchers interested in autophagy in PDAC and welcome researchers all over the world to go further based on our findings. Second, the outcome of Genome‐wide association studies (GWAS) is another kind of multi‐dimensional data hard to be included in our clustering analysis. By detecting low penetrance variants by GWAS, some genetic susceptibility of PDAC could be explained. However, although some large PDAC consortiums such as PanScan, PanC4, and PANDoRA have been finished to detect possible loci, only small number of loci reaching the genome‐wide significance. Ye Lu, et al.^58^, ^59^ have tried to explore more potential loci and miRNA‐related loci by secondary analysis under a recessive model, but many of the meta‐analysis results did not reach the genome‐wide statistical significance. The false negative outcomes may come from the high significance threshold in meta‐analysis, indicating the necessity of the development of new integrating method rather than simply pooled analysis. Exclusion of GWAS outcomes in multi‐omics analyses may be a lost part of the whole regulation network, but how to integrate loci estimation in genome‐wide level and with other omics data are still a huge challenge. We are interested in cooperating with other multi‐omics analyses experts and developing new approaches.

In conclusion, we constructed an autophagy‐related mRNA/miRNA/TF/Immune cells network based on a 10 state‐of art algorithm multi‐omics analysis, and screened the drug sensitivity dataset for detecting potential signal pathway which might be possible autophagy modulators' targets.

## AUTHOR CONTRIBUTIONS

CYH and WCH came up with the idea. CYH and MJL finished all the analyses. LXF maintained the code. CYH and LX drew pictures. CYH wrote the article and LX polished it.

## CONFLICT OF INTEREST

The authors declare that the research was conducted in the absence of any commercial or financial relationships that could be construed as a potential conflict of interest.

## ETHICS APPROVAL STATEMENT

All data of this study were public and required no ethical approval.

## Supporting information


Table S1
Table S2
Table S3
Table S4
Table S5
Table S6

## Data Availability

All original data used to be data‐mining are available at TCGA (https://portal.gdc.cancer.gov/projects/TCGA‐PAAD) and GEO (https://www.ncbi.nlm.nih.gov/geo). The codes of MOVICS package we used are available on Github (https://xlucpu.github.io/MOVICS/MOVICS‐VIGNETTE.html).
