# Supplementary material for: Clustering analysis revealed the autophagy classification and potential autophagy regulators' sensitivity of pancreatic cancer based on multi‐omics data
Source: Cancer Med. 2022 Jun 9;12(1):733–46. doi: 10.1002/cam4.4932 (PMC9844610; doi:10.1002/cam4.4932)
Supplement: Supplementary file 1 — Table S1 Table S2 Table S3 Table S4 Table S5 Table S6 [file CAM4-12-733-s001.docx]

| Gene (mRNA) | HR | Z | P-value | lower | upper |
| --- | --- | --- | --- | --- | --- |
| MICAL1 | 0.44 | -5.01 | 5.47E-07 | 0.32 | 0.61 |
| SLC26A11 | 0.39 | -4.94 | 7.88E-07 | 0.27 | 0.56 |
| TTC13 | 0.39 | -4.65 | 3.26E-06 | 0.27 | 0.58 |
| INTS6L | 0.43 | -4.62 | 3.75E-06 | 0.31 | 0.62 |
| CAMTA2 | 0.38 | -4.61 | 3.96E-06 | 0.25 | 0.57 |
| ST3GAL2 | 0.43 | -4.59 | 4.49E-06 | 0.30 | 0.62 |
| NSUN6 | 0.38 | -4.48 | 7.55E-06 | 0.24 | 0.58 |
| SLC25A35 | 0.33 | -4.47 | 7.92E-06 | 0.20 | 0.54 |
| LIPT1 | 0.34 | -4.46 | 8.36E-06 | 0.22 | 0.55 |
| MFNG | 0.52 | -4.37 | 1.26E-05 | 0.39 | 0.70 |
| GPSM3 | 0.55 | -4.29 | 1.76E-05 | 0.41 | 0.72 |
| KIAA0513 | 0.44 | -4.28 | 1.83E-05 | 0.31 | 0.64 |
| WASHC2C | 0.42 | -4.28 | 1.84E-05 | 0.28 | 0.63 |
| TRAF5 | 0.48 | -4.26 | 2.04E-05 | 0.35 | 0.68 |
| TRAF1 | 0.55 | -4.20 | 2.67E-05 | 0.41 | 0.73 |
| WDR37 | 0.41 | -4.20 | 2.69E-05 | 0.27 | 0.62 |
| FIG4 | 0.38 | -4.19 | 2.76E-05 | 0.24 | 0.60 |
| ZMAT1 | 0.57 | -4.19 | 2.83E-05 | 0.44 | 0.74 |
| UPK2 | 1.28 | 4.18 | 2.96E-05 | 1.14 | 1.44 |
| DENND4B | 0.43 | -4.17 | 2.98E-05 | 0.29 | 0.64 |
| **Table S1.** top 20 significant mRNA got elites from huge dimensional data based on univariate Cox proportional hazard regression. | | | | | |

| miRNA | HR | Z | P-value | lower | upper |
| --- | --- | --- | --- | --- | --- |
| hsa-miR-3613-5p | 0.28 | -3.43 | 0.00 | 0.14 | 0.58 |
| hsa-miR-140-5p | 0.05 | -3.24 | 0.00 | 0.01 | 0.32 |
| hsa-miR-146a-5p | 0.18 | -3.17 | 0.00 | 0.06 | 0.52 |
| hsa-miR-590-3p | 0.30 | -3.09 | 0.00 | 0.14 | 0.65 |
| hsa-miR-145-3p | 0.07 | -3.08 | 0.00 | 0.01 | 0.37 |
| hsa-miR-374a-3p | 0.02 | -2.99 | 0.00 | 0.00 | 0.26 |
| hsa-miR-218-5p | 0.20 | -2.95 | 0.00 | 0.07 | 0.59 |
| hsa-miR-653-5p | 0.34 | -2.86 | 0.00 | 0.16 | 0.71 |
| hsa-miR-98-5p | 0.03 | -2.76 | 0.01 | 0.00 | 0.38 |
| hsa-miR-29c-5p | 0.23 | -2.62 | 0.01 | 0.08 | 0.69 |
| hsa-miR-126-3p | 0.05 | -2.53 | 0.01 | 0.00 | 0.51 |
| hsa-miR-195-5p | 0.24 | -2.52 | 0.01 | 0.08 | 0.73 |
| hsa-let-7b-5p | 375.97 | 2.50 | 0.01 | 3.62 | 39000.44 |
| hsa-miR-29b-2-5p | 0.18 | -2.49 | 0.01 | 0.05 | 0.69 |
| hsa-miR-126-5p | 0.11 | -2.43 | 0.02 | 0.02 | 0.65 |
| hsa-miR-30e-5p | 0.01 | -2.42 | 0.02 | 0.00 | 0.40 |
| hsa-miR-222-5p | 2.69 | 2.37 | 0.02 | 1.19 | 6.08 |
| hsa-miR-374b-5p | 0.13 | -2.37 | 0.02 | 0.02 | 0.70 |
| hsa-miR-221-3p | 7.29 | 2.32 | 0.02 | 1.36 | 39.21 |
| **Table S2.** top 20 significant miRNA got elites from huge dimensional data based on univariate Cox proportional hazard regression. | | | | | |

| lncRNA | HR | Z | P-value | lower | upper |
| --- | --- | --- | --- | --- | --- |
| MIR3142HG | 0.55 | -4.27 | 1.96E-05 | 0.42 | 0.72 |
| AL358472.2 | 0.39 | -4.12 | 3.73E-05 | 0.25 | 0.61 |
| AC022182.1 | 0.22 | -4.07 | 4.77E-05 | 0.11 | 0.46 |
| FLVCR1-AS1 | 0.54 | -3.89 | 0.000100576 | 0.39 | 0.74 |
| AC010719.1 | 0.64 | -3.82 | 0.000134797 | 0.51 | 0.81 |
| AC012236.1 | 0.61 | -3.76 | 0.000169649 | 0.47 | 0.79 |
| AC068620.3 | 0.48 | -3.74 | 0.000182401 | 0.32 | 0.70 |
| AC092119.2 | 0.57 | -3.74 | 0.000182456 | 0.43 | 0.77 |
| AC009093.6 | 0.57 | -3.74 | 0.000187369 | 0.42 | 0.76 |
| LINC01160 | 0.35 | -3.73 | 0.000191352 | 0.20 | 0.61 |
| SOCS2-AS1 | 0.41 | -3.70 | 0.000212652 | 0.26 | 0.66 |
| AL157392.3 | 0.42 | -3.65 | 0.000258326 | 0.26 | 0.67 |
| AC068987.4 | 0.43 | -3.63 | 0.000281569 | 0.27 | 0.68 |
| AC007066.2 | 0.41 | -3.62 | 0.000294459 | 0.25 | 0.67 |
| TRAM2-AS1 | 0.43 | -3.61 | 0.00031181 | 0.27 | 0.68 |
| AC024075.2 | 0.50 | -3.60 | 0.000322288 | 0.34 | 0.73 |
| AC068580.2 | 1.37 | 3.59 | 0.000335023 | 1.16 | 1.64 |
| GATA6-AS1 | 0.69 | -3.54 | 0.000397826 | 0.56 | 0.85 |
| Z97832.2 | 0.38 | -3.53 | 0.000418933 | 0.23 | 0.65 |
| **Table S3.** top 20 significant lncRNA got elites from huge dimensional data based on univariate Cox proportional hazard regression. | | | | | |

| meth | HR | Z | P-value | lower | upper |
| --- | --- | --- | --- | --- | --- |
| cg25087487 | 1.06E+03 | 4.60E+00 | 4.25E-06 | 5.45E+01 | 2.07E+04 |
| cg26546557 | 1.53E+02 | 4.39E+00 | 1.16E-05 | 1.61E+01 | 1.44E+03 |
| cg18701590 | 6.23E+08 | 4.30E+00 | 1.73E-05 | 6.07E+04 | 6.40E+12 |
| cg13361843 | 2.22E+01 | 4.18E+00 | 2.97E-05 | 5.18E+00 | 9.50E+01 |
| cg11174851 | 4.39E+01 | 4.03E+00 | 5.47E-05 | 6.99E+00 | 2.75E+02 |
| cg06000963 | 3.33E+01 | 4.00E+00 | 6.24E-05 | 5.98E+00 | 1.85E+02 |
| cg15235096 | 2.09E+05 | 3.97E+00 | 7.30E-05 | 4.91E+02 | 8.88E+07 |
| cg00888162 | 2.79E+06 | 3.94E+00 | 8.18E-05 | 1.73E+03 | 4.49E+09 |
| cg24950336 | 1.65E+05 | 3.93E+00 | 8.35E-05 | 4.15E+02 | 6.55E+07 |
| cg01971137 | 7.92E+00 | 3.92E+00 | 8.74E-05 | 2.82E+00 | 2.23E+01 |
| cg00803804 | 7.02E+12 | 3.92E+00 | 8.79E-05 | 2.67E+06 | 1.85E+19 |
| cg23994917 | 2.06E+02 | 3.86E+00 | 1.15E-04 | 1.37E+01 | 3.09E+03 |
| cg05798608 | 1.61E+11 | 3.83E+00 | 1.27E-04 | 2.98E+05 | 8.69E+16 |
| cg04349810 | 4.86E+29 | 3.83E+00 | 1.29E-04 | 3.09E+14 | 7.64E+44 |
| cg04264018 | 2.32E+09 | 3.79E+00 | 1.48E-04 | 3.37E+04 | 1.60E+14 |
| cg10677697 | 8.62E+00 | 3.77E+00 | 1.64E-04 | 2.81E+00 | 2.64E+01 |
| cg00397851 | 1.07E+02 | 3.77E+00 | 1.66E-04 | 9.41E+00 | 1.22E+03 |
| cg10501085 | 2.57E+01 | 3.75E+00 | 1.76E-04 | 4.71E+00 | 1.40E+02 |
| cg13534424 | 1.13E+03 | 3.74E+00 | 1.81E-04 | 2.85E+01 | 4.47E+04 |
| **Table S4.** top 20 significant methylations got elites from huge dimensional data based on univariate Cox proportional hazard regression. | | | | | |

|  | GAPDH | HSPA5 | MAP1LC3B | BCL2L1 | SQSTM1 | MAPK3 | RAB5A | EIF2S1 | CTSD | RAB7A | RAB11A | DDIT3 | VEGFA | RELA | ERBB2 | HSP90AB1 | CDKN2A | BAG3 | RHEB | FADD | B cells naive | B cells memory | T cells CD8 | T cells CD4 memory resting | Macrophages M0 |
| --- | --- | --- | --- | --- | --- | --- | --- | --- | --- | --- | --- | --- | --- | --- | --- | --- | --- | --- | --- | --- | --- | --- | --- | --- | --- |
| GAPDH | 1 | -0.05 | -0.09 | 0.04 | -0.06 | -0.08 | -0.11 | 0.03 | -0.04 | 0.03 | 0.11 | -0.02 | 0.02 | -0.01 | -0.05 | -0.08 | 0.06 | -0.05 | 0.01 | 0.03 | 0.11 | 0.28 | 0.06 | -0.04 | 0.01 |
| HSPA5 | -0.05 | 1 | 0.25 | 0.01 | 0.27 | 0.11 | 0.15 | 0.13 | 0.01 | 0.25 | 0.15 | 0.38 | 0.18 | 0.04 | -0.03 | 0.43 | 0.03 | 0.47 | 0.07 | 0.01 | -0.04 | -0.14 | -0.12 | 0.16 | -0.11 |
| MAP1LC3B | -0.09 | 0.25 | 1 | 0.3 | 0.33 | 0.1 | 0.41 | 0.24 | 0.1 | 0.43 | 0.22 | 0.43 | 0.16 | 0.26 | -0.05 | 0.26 | 0.13 | 0.28 | 0.22 | 0.03 | 0.02 | -0.03 | -0.04 | -0.1 | -0.03 |
| BCL2L1 | 0.04 | 0.01 | 0.3 | 1 | 0 | -0.25 | 0.18 | 0.2 | 0.03 | 0.29 | -0.08 | -0.16 | -0.07 | 0 | -0.01 | 0.04 | -0.08 | 0.02 | 0.28 | 0.03 | 0.1 | 0.21 | -0.01 | -0.11 | 0.06 |
| SQSTM1 | -0.06 | 0.27 | 0.33 | 0 | 1 | 0.35 | 0.16 | 0.08 | 0.24 | 0.36 | 0.25 | 0.47 | 0.18 | 0.52 | 0.16 | 0.2 | 0.29 | 0.29 | 0.03 | 0.27 | -0.11 | -0.13 | -0.15 | 0.02 | 0.15 |
| MAPK3 | -0.08 | 0.11 | 0.1 | -0.25 | 0.35 | 1 | 0.19 | 0.06 | 0.09 | 0.15 | 0.41 | 0.21 | 0.09 | 0.48 | 0.16 | 0.09 | 0.09 | 0.07 | -0.06 | 0.33 | -0.17 | 0.01 | -0.28 | 0.18 | 0.1 |
| RAB5A | -0.11 | 0.15 | 0.41 | 0.18 | 0.16 | 0.19 | 1 | 0.41 | -0.01 | 0.46 | 0.37 | 0.1 | 0.12 | 0.23 | 0.03 | 0.08 | 0.1 | 0.08 | 0.14 | 0.05 | -0.19 | -0.06 | -0.34 | 0.12 | 0.05 |
| EIF2S1 | 0.03 | 0.13 | 0.24 | 0.2 | 0.08 | 0.06 | 0.41 | 1 | -0.07 | 0.41 | 0.28 | 0.04 | 0.02 | 0.15 | 0.08 | 0.19 | 0.1 | 0.03 | 0.2 | 0.25 | -0.05 | -0.04 | -0.11 | -0.07 | 0.14 |
| CTSD | -0.04 | 0.01 | 0.1 | 0.03 | 0.24 | 0.09 | -0.01 | -0.07 | 1 | 0.24 | -0.04 | 0.07 | 0.04 | 0.26 | 0.05 | -0.1 | 0.21 | 0.09 | 0.21 | 0.26 | -0.01 | -0.05 | -0.09 | -0.31 | 0.22 |
| RAB7A | 0.03 | 0.25 | 0.43 | 0.29 | 0.36 | 0.15 | 0.46 | 0.41 | 0.24 | 1 | 0.31 | 0.23 | 0.11 | 0.3 | 0.14 | 0.24 | 0.14 | 0.3 | 0.32 | 0.22 | -0.08 | -0.06 | -0.13 | -0.01 | 0.07 |
| RAB11A | 0.11 | 0.15 | 0.22 | -0.08 | 0.25 | 0.41 | 0.37 | 0.28 | -0.04 | 0.31 | 1 | 0.15 | 0.12 | 0.24 | 0.19 | 0.18 | 0.05 | 0.14 | -0.02 | 0.28 | -0.13 | 0.04 | -0.12 | 0.16 | -0.01 |
| DDIT3 | -0.02 | 0.38 | 0.43 | -0.16 | 0.47 | 0.21 | 0.1 | 0.04 | 0.07 | 0.23 | 0.15 | 1 | 0.41 | 0.29 | -0.01 | 0.28 | 0.14 | 0.37 | 0.2 | -0.07 | -0.05 | -0.11 | 0.02 | -0.04 | 0.07 |
| VEGFA | 0.02 | 0.18 | 0.16 | -0.07 | 0.18 | 0.09 | 0.12 | 0.02 | 0.04 | 0.11 | 0.12 | 0.41 | 1 | 0.25 | 0.1 | 0.22 | 0.2 | 0.19 | -0.02 | -0.05 | -0.11 | -0.1 | -0.11 | -0.01 | 0.14 |
| RELA | -0.01 | 0.04 | 0.26 | 0 | 0.52 | 0.48 | 0.23 | 0.15 | 0.26 | 0.3 | 0.24 | 0.29 | 0.25 | 1 | 0.09 | -0.02 | 0.24 | 0.29 | 0.11 | 0.51 | -0.08 | -0.04 | -0.2 | 0.02 | 0.2 |
| ERBB2 | -0.05 | -0.03 | -0.05 | -0.01 | 0.16 | 0.16 | 0.03 | 0.08 | 0.05 | 0.14 | 0.19 | -0.01 | 0.1 | 0.09 | 1 | -0.02 | -0.02 | -0.01 | -0.05 | 0.04 | -0.08 | 0.04 | -0.16 | -0.06 | 0.19 |
| HSP90AB1 | -0.08 | 0.43 | 0.26 | 0.04 | 0.2 | 0.09 | 0.08 | 0.19 | -0.1 | 0.24 | 0.18 | 0.28 | 0.22 | -0.02 | -0.02 | 1 | 0.18 | 0.35 | 0.02 | 0.01 | -0.03 | -0.14 | 0 | 0.03 | -0.03 |
| CDKN2A | 0.06 | 0.03 | 0.13 | -0.08 | 0.29 | 0.09 | 0.1 | 0.1 | 0.21 | 0.14 | 0.05 | 0.14 | 0.2 | 0.24 | -0.02 | 0.18 | 1 | 0.09 | 0.18 | 0.19 | -0.05 | -0.08 | -0.08 | -0.13 | 0.09 |
| BAG3 | -0.05 | 0.47 | 0.28 | 0.02 | 0.29 | 0.07 | 0.08 | 0.03 | 0.09 | 0.3 | 0.14 | 0.37 | 0.19 | 0.29 | -0.01 | 0.35 | 0.09 | 1 | 0.23 | 0.04 | -0.05 | -0.06 | -0.08 | 0 | 0.01 |
| RHEB | 0.01 | 0.07 | 0.22 | 0.28 | 0.03 | -0.06 | 0.14 | 0.2 | 0.21 | 0.32 | -0.02 | 0.2 | -0.02 | 0.11 | -0.05 | 0.02 | 0.18 | 0.23 | 1 | 0.12 | 0.03 | 0.03 | 0.01 | -0.2 | 0.09 |
| FADD | 0.03 | 0.01 | 0.03 | 0.03 | 0.27 | 0.33 | 0.05 | 0.25 | 0.26 | 0.22 | 0.28 | -0.07 | -0.05 | 0.51 | 0.04 | 0.01 | 0.19 | 0.04 | 0.12 | 1 | 0 | -0.04 | -0.09 | -0.02 | 0.06 |
| B cells naive | 0.11 | -0.04 | 0.02 | 0.1 | -0.11 | -0.17 | -0.19 | -0.05 | -0.01 | -0.08 | -0.13 | -0.05 | -0.11 | -0.08 | -0.08 | -0.03 | -0.05 | -0.05 | 0.03 | 0 | 1 | 0.21 | 0.29 | -0.22 | -0.19 |
| B cells memory | 0.28 | -0.14 | -0.03 | 0.21 | -0.13 | 0.01 | -0.06 | -0.04 | -0.05 | -0.06 | 0.04 | -0.11 | -0.1 | -0.04 | 0.04 | -0.14 | -0.08 | -0.06 | 0.03 | -0.04 | 0.21 | 1 | 0.1 | -0.1 | -0.04 |
| T cells CD8 | 0.06 | -0.12 | -0.04 | -0.01 | -0.15 | -0.28 | -0.34 | -0.11 | -0.09 | -0.13 | -0.12 | 0.02 | -0.11 | -0.2 | -0.16 | 0 | -0.08 | -0.08 | 0.01 | -0.09 | 0.29 | 0.1 | 1 | -0.15 | -0.5 |
| T cells CD4 memory resting | -0.04 | 0.16 | -0.1 | -0.11 | 0.02 | 0.18 | 0.12 | -0.07 | -0.31 | -0.01 | 0.16 | -0.04 | -0.01 | 0.02 | -0.06 | 0.03 | -0.13 | 0 | -0.2 | -0.02 | -0.22 | -0.1 | -0.15 | 1 | -0.38 |
| Macrophages M0 | 0.01 | -0.11 | -0.03 | 0.06 | 0.15 | 0.1 | 0.05 | 0.14 | 0.22 | 0.07 | -0.01 | 0.07 | 0.14 | 0.2 | 0.19 | -0.03 | 0.09 | 0.01 | 0.09 | 0.06 | -0.19 | -0.04 | -0.5 | -0.38 | 1 |

**Table S5. Correlation coefficients between autophagy genes and immune cell population.**

| Drugs | IC50 in CS1 | IC50 in CS2 | P value |
| --- | --- | --- | --- |
| Imatinib | 3.37 | 3.33 | 9.10E-07 |
| bryostatin1 | -2.48 | -2.625 | 2.70E-08 |
| AUY922 | -2.7 | -3.1 | 5.30E-05 |
| GW-2580 | 5.5 | 5.4 | 2.20E-03 |
| LFM-A13 | 5 | 4.8 | 4.40E-03 |
| Bleomycin | 1.5 | 1.1 | 3.00E-02 |
| Bexarotene | 3.5 | 3.4 | 3.00E-02 |
| listinib | 2.14 | 2.14 | 8.40E-03 |
| Thapsigargin | -4.1 | -3.9 | 1.70E-02 |
| BAY 61-3606 | 2.8 | 2 | 1.30E-09 |
| IPA-3 | 4.8 | 4 | 3.20E-05 |
| Embelin | 2.8 | 2.75 | 1.10E-02 |
| OSU-03012 | 1.9 | 1.75 | 3.90E-03 |
| fti-277 | 2.4 | 2.3 | 3.90E-02 |
| jq1 | 0.6 | 0.4 | 2.60E-02 |
| HG-6-64-1 | 1.4 | 1 | 3.60E-06 |
| PF-562271 | 1.9 | 1.8 | 1.40E-01 |
| JNK-9L | -0.25 | -0.3 | 1.40E-02 |
| AP-24534 | 0.8 | 0.6 | 1.90E-08 |
| cp466722 | 3.1 | 298 | 2.30E-05 |
| QS11 | 3.5 | 3.1 | 7.20E-04 |
| Bicalutamide | 2.9 | 2.5 | 1.40E-05 |
| NSC-87877 | 5 | 4.9 | 2.70E-03 |
| Gemcitabine | -2.1 | -1.9 | 1.90E-02 |
| Etoposide | 2 | 1.9 | 5.00E-02 |
| Doxorubicin | -1.5 | -1.4 | 3.70E-02 |
| GSK269962A | 2.7 | 2.4 | 1.20E-04 |
| Lapatinib | 2.4 | 2.6 | 4.00E-07 |
| Salubrinal | 4 | 3.9 | 1.00E-03 |
| XMD8-85 | 2.8 | 2.9 | 4.10E-03 |
| Bortezomib | -5.8 | -5.6 | 1.70E-04 |
| TGX221 | 4.3 | 4.4 | 1.70E-06 |
| KIN001-135 | 4.2 | 4.1 | 7.00E-08 |
| Parthenolide | 3.7 | 3.5 | 1.70E-05 |
| MS-275 | 1.5 | 1.25 | 1.20E-04 |
| GW843682X | -3 | -2.5 | 9.70E-04 |
| A443654 | -0.75 | -0.5 | 4.90E-03 |
| Pyrimethamine | 3.7 | 3.5 | 2.30E-05 |
| CMK | 2.5 | 2.3 | 6.80E-04 |
| BMS-509744 | 2.7 | 3 | 1.60E-04 |
| bl-2536 | -2 | -1.7 | 2.30E-03 |
| wz-1-84 | 4.1 | 4.2 | 2.40E-08 |
| WH-4-023 | 2.5 | 2.7 | 1.50E-01 |
| A-770041 | 2 | 2.5 | 4.50E-06 |
| CGP-082966 | 2.7 | 3.1 | 3.70E-04 |
| gnf-2 | 2.5 | 2.6 | 1.70E-10 |
| Dasatinib | 0.5 | 1.1 | 4.60E-10 |
| z-llnle-cho | 0.7 | 0.8 | 1.30E-04 |
| S-Trityl-L-cysteine | 1.3 | 1.1 | 2.80E-05 |
| Saracatinib | 2.3 | 2.4 | 1.10E-03 |
| Crizotinib | 2.8 | 2.6 | 1.30E-07 |
| Sorafenib | 2.5 | 2.4 | 1.00E-04 |
| AZ628 | 1.8 | 1.7 | 1.10E-02 |
| MH-132 | 0 | 0.1 | 1.80E-01 |
| pha-665752 | 2.9 | 2.9 | 3.90E-04 |
| Rapamycin | -2.5 | -2 | 8.90E-03 |

**Table S6.** **Table listing IC50 of CS1, CS2 and their difference and p-value for each drug.**
